# Physicochemical properties, antioxidant capacities, and metal contents of virgin coconut oil produced by wet and dry processes

**DOI:** 10.1002/fsn3.671

**Published:** 2018-05-23

**Authors:** Nurul Aqilah A. Ghani, Amy‐Arniza Channip, Phoebe Chok Hwee Hwa, Fairuzeta Ja'afar, Hartini M. Yasin, Anwar Usman

**Affiliations:** ^1^ Faculty of Science Department of Chemistry Universiti Brunei Darussalam Gadong Negara Brunei Darussalam

**Keywords:** antioxidants, fatty acid content, lauric acid, physicochemical properties, virgin coconut oil

## Abstract

Different from cooking oils which contain long‐chain fatty acids, virgin coconut oil (VCO) has high medium‐chain fatty acids, making it a potential functional food which can provide some health benefits. In this study, our objective is to investigate the physicochemical properties, antioxidant capacity, and metal contents of the VCO extracted through four different processing methods: chilling and centrifugation; fermentation; direct micro expelling‐oven dried; and direct micro expelling‐sun‐dried processes. We found that the physicochemical properties, including moisture content, refractive index, viscosity, iodine value, saponification value, peroxide value, free fatty acid, and fatty acid content, of all the VCO conform to the Asian and Pacific Coconut Community (APCC) standard. All of the VCO predominantly contains lauric acid which is in the range of 48.40%–52.84% of the fatty acid content. The total phenolic content and DPPH radical‐scavenging activity (IC_50_) of the VCO was obtained to be in the range of 1.16–12.54 mg gallic acid equivalents (GAE)/g and 7.49–104.52 mg/ml, respectively, and the metal contents in the VCO were within the acceptable range of the recommended APCC limit. These findings ensure good quality and safety assurance of the VCO produced from the coconut grown in Brunei Darussalam through the different processing methods.

## 1 INTRODUCTION

Virgin coconut oil (VCO) is an edible oil obtained from the milk of fresh and matured kernel of the coconut (*Cocos nucifera* L.) (Marina, Che Man, & Amin, 2009), a tropical plant belonging to the *Arecaceae* (palm) family. VCO is colorless with the aroma of fresh coconut, and it has been largely consumed for many purposes in cooking, bakery, confectionary, infant foods, and cosmetics. In cosmetics, VCO is utilized as a substance to enhance beauty, to promote the growth of hairs, and to improve and moisturize skin. Another emerging application of VCO is in the health supplement area, due to the health benefits of medium‐chain fatty acids (MCFA) contained in VCO. MCFA such as lauric, myristic, palmitic, capric, stearic, oleic, and linoleic acids are easily digestible (DebMandal & Mandal, 2011). The lauric acid component in VCO, in particular, was reported to show potential for anti‐obesity treatments (Assunção, Ferreira, Dos Santos, Cabral, & Florêncio, 2009; Nevin & Rajamohan, 2004; St‐Onge & Jones, 2002). Moreover, MCFA have some specific functional and nutritional properties, including antiviral, antibacterial, antiplaque, antiprotozoal, healing, anti‐inflammatory, and anti‐obesity effects (German & Dillard, 2004; Gopala Krishna, Raj, Bhatnagar, Prasanth, & Chandrashekar, 2010). Therefore, owing to the essential MCFA content, VCO exhibits some advantages to heal several minor illnesses such as diarrhea, skin inflammations, gastrointestinal problems, minor cuts, injuries, and swelling (Nevin & Rajamohan, 2010). These properties promote further uses of VCO; for instance, VCO has been recognized as a multipurpose nutrient supplement due to the nutritional and medicinal benefits of its MCFA, vitamins, amino acids, antioxidants, antimicrobial, and antiviral compounds (Kabara, 1984; Nevin & Rajamohan, 2006).

Virgin coconut oil is produced by several methods which can be generally categorized into wet and dry methods (Bawalan & Chapman, 2006). In the wet method, VCO is directly extracted from the coconut meat/kernel by either chilling and centrifugation, fermentation, enzymatic, pH method, or any of these combinations to destabilize the coconut milk emulsion without the drying process (Raghavendra & Raghavarao, 2010). In contrast, for the dry method, the kernel is dried by controlled heating to remove the moisture, preventing microbial invasion from occurring. VCO is obtained by pressing the dried kernel mechanically. The yield of VCO strongly depends not only on the extraction methods but also on several factors, including time of harvesting, age of coconut, location of plantation, and the age of copra before the extraction (Carandang, 2008). An important deciding factor in the assessment of the quality of VCO is its physicochemical properties, such as moisture content, fatty acid content, free fatty acid content, iodine value, peroxide value, saponification value, and viscosity. In addition, total phenolic content, and antioxidant capacity and the metal composition in VCO are known to affect its rate of oxidation, nutritional value, preservation properties, and shelf life (Murillo et al., 1999). Several reviews on the quality of VCO have been reported (Amri, 2011; Belitz & Grosch, 1999; Gopala Krishna et al., 2010; Marina, Che Man, & Amin, 2009), and its physicochemical properties have been standardized by the Asian and Pacific Coconut Community (APCC, 2009). However, not all of the quality and nutritional characteristics of VCO in the Asian and Pacific area are reported. In particular, VCO extracted in the coconut industries in Brunei Darussalam has never been reported. It is therefore necessary to determine the quality of VCO produced in the area.

This work focuses on the physicochemical properties of VCO extracted by different methods to meet the quality of VCO according to the APCC standard (APCC (Asian Pacific Coconut Community), 2009). The extraction of VCO was carried out using both wet and dry processes, including chilling and centrifugation (C&C), fermentation (FER), and direct micro expelling (DME) methods. All these processes are known to be efficient and quick to produce VCO with the high heat stability. To evaluate the antioxidant potential of the VCO, its total phenolic content, total flavonoid content, and antioxidant capacity were determined using chemical assays. The metal contents in the VCO were also investigated. This study reports the physicochemical and quality characteristics of the VCO, including moisture content, fatty acid content, free fatty acid content, iodine value, peroxide value, saponification value, refractive index, and viscosity, as well as total phenolic content, total flavonoid content, antioxidant capacity, and metal contents in the VCO produced in Brunei Darussalam.

## 2 MATERIALS AND METHODS

### 2.1 Raw materials

Freshly harvested, mature coconuts aged more than 12 months were obtained from local markets in Tutong and Brunei‐Muara district in Brunei Darussalam. The variety of the coconut was Malayan tall dwarf (MTD). Only coconuts which had not sprouted were selected, and their milk was taken out from the coconut fruits. The coconut milk was purified by a local company, IMBRU Essential Oils. It then was further processed into VCO using C&C, FER, and DME methods as described below.

The solvents used for the physiochemical analyses of VCO are methanol, ethanol, acetic acid, hydrochloric acid, hexane, and Wijs solution. The chemicals used to evaluate the antioxidant capacity and total phenolic content (TPC) are 2,2‐diphenyl‐1‐picrylhydrazyl radical (DPPH), Folin–Ciocalteu reagent, hydrochloric acid (HCl), potassium iodide (KI), nitric acid (HNO_3_), ethanol, sodium thiosulfate (Na_2_S_2_O_3_), sodium hydroxide (NaOH), potassium hydroxide (KOH), acetic acid (CH_3_COOH), hydrogen peroxide (H_2_O_2_), chloroform (CHCl_3_), and sodium carbonate (Na_2_CO_3_). All the solvents and chemicals were obtained from Sigma‐Aldrich Co. (St. Louis, MO), Merck (Darmstadt‐Germany) or RCl Labscan (Thailand), and they were used as received without any further purification.

### 2.2 Extraction of VCO

#### 2.2.1 Wet process

The coconut milk was obtained according to the protocols outlined by Neela and Prasad (2012). The VCO extracted using integrated wet processes was performed according to reported procedures (Nur Arbainah, 2012) with slight modifications. The solid endosperm of mature coconut was de‐husked, collected, and grated. The water from the inner cavity was disposed. It was then squeezed and filtered through cheesecloth to obtain the coconut milk.

#### 2.2.2 C&C method

For C&C, VCO was extracted according to the reported procedures (Raghavendra & Raghavarao, 2010; Seow & Gwee, 1997) with some modifications. Here, the coconut milk was chilled to below 4°C and mixed using a rotator for 15 min. The upper layer of cream was separated from the water layer, and it was then removed for thawing in a water bath at 50°C. This was followed by centrifugation at 6,000 rpm for 45 min to separate the VCO further from aqueous layer.

#### 2.2.3 FER method

In the FER method, the coconut milk was left undisturbed to ferment naturally at room temperature. After keeping for 72 hr, the layers of VCO and water in the mixture were separated by centrifugation at 6,000 rpm for 45 min. The VCO which was the upper layer was simply drawn off.

### 2.3 Dry process

The VCO was extracted using dry process according to the DME method given by Asian Pacific Coconut Community (APCC, 2009) with some minor modifications. In this dry process, the kernel of the coconut was heated under controlled conditions, depending on the oven‐dry or sun‐dry process, to remove its moisture content, while preventing any microbial invasion from occurring. Subsequently, the dried kernel was pressed mechanically to obtain its oil. In this current study, we have applied two DME approaches, namely oven‐dried (DME‐OD) and sun‐dried (DME‐SD), to remove the moisture content in the grated coconut meat before the oil is being extracted. For the DME‐OD method, the grated coconut meat was dried in an oven operating at a temperature of 40°C for 4 hr. For the DME‐SD method, the grated coconut meat was dried under sunshine for about 3–4 hr. Then, the dried grated coconut meat was pressed with a modified mechanical jack to produce the VCO. The separated and purified VCO were refrigerated until further use.

### 2.4 Determination of physicochemical properties

#### 2.4.1 Moisture content

Determination of moisture content (MC) in the VCO was based on the American Oil Chemists Society (Firestone, 2009) method. About 5.0 g of the VCO sample was placed into a pre‐heated and pre‐weighed crucible with lid, and then heated at 105°C for at least 24 hr. The sample was then placed in a desiccator and allowed to cool down to room temperature. The crucible containing the VCO was then re‐weighed. The moisture and volatile content was calculated using the following formula;

(1) $$\text{MC =}\frac{\text{(initial}\;\text{weight ‐ final}\;\text{weight)}}{\text{initial}\;\text{weight}}\times 100\%$$

#### 2.4.2 Refractive index

The refractive index (RI) of the VCO samples were measured using a precision Abbé refractometer (Bellingham & Stanley, U.K.) having a measuring range of refractive index of 1.300–1.700 with the accuracy within ±0.0002. A thin layer of VCO was sandwiched between illuminating and refracting prisms. The sample was then illuminated with monochromatic light from sodium vapor lamp, and the RI was recorded.

#### 2.4.3 Free fatty acid

The free fatty acid (FFA) content of the VCO was measured according to the standard Association of Official Agricultural Chemists (AOAC) method (Horwitz, 2000). About 7.05 g of each VCO sample was mixed with 2 ml phenolphthalein solution and a few drops of 0.1 M NaOH; 50 ml of ethanol was then added to the solution and vigorously shaken until a permanent faint pink solution remained, which was then titrated with 0.25 N NaOH. The volume of spent NaOH was recorded and represented as *S*. For the control measurement, the titration step was repeated on a blank solution without the VCO and the volume of spent NaOH is represented as *B*. The percentage of FFA (% FFA) was calculated using

(2) $$\%\text{FFA}=\frac{(B-S)\text{ml}\;\text{of}\;\text{NaOH}\times N\times 56}{1.99\times \text{weight}\;\text{of}\;\text{sample}}$$

where N is the normality of titer, NaOH.

#### 2.4.4 Fatty acid methyl ester

The fatty acid methyl ester (FAME) was extracted according to the AOAC method (Horwitz, 2000). Approximately 50 mg of VCO was dissolved in 4 ml of 0.5 mol/L methanolic HCl. The solution was mixed thoroughly, followed by incubation at 50°C for 4 hr and cooling to room temperature. FAME was purified using 10 ml of hexane, and the clear upper layer containing FAME was then passed through anhydrous Na_2_SO_4_ for drying. The extracted FAME was identified using gas chromatography following the protocols outlined by Moigradean, Poiama, Alda, and Gogoasa (2013). The composition of FAME was evaluated using a gas chromatography‐mass spectrometer (GC‐MS) QP 2010 (Shimadzu, Japan) equipped with a split/split less injector. The separation of the compounds was performed on a DB‐5 ms column (length 30 m, diameter 0.25 mm, and thickness 0.25‐μm film). Helium was used at flow rate of 1.00 ml/min and a split ratio of 100.0. The injector temperature was 250°C. The oven temperature was held at 60°C for 10 min, and it was increased to 140°C at a rate of 10°C/min and the final temperature was held for 10 min. The temperature was then further increased to 250°C at a rate of 7°C/min and held at this final temperature for 10 min. Mass‐selective detector conditions were set at capillary direct interface temperature of 230°C, ionization energy of 70 eV; and full‐scan mode with a mass range of 40–850 amu. The FAME in the VCO were identified by matching the retention indexes and mass spectra of the unknown compounds with those of standard compounds. The weight fractions of the FAMEs were measured based on the percentage represented by the area of corresponding peak relative to the sum of the area of all peaks.

#### 2.4.5 Iodine value

Iodine value (IV) of the VCO was determined using Wijs method (AOCS, 2004). Approximately 3.0 g of VCO was mixed with 20 ml cyclohexane to dissolve the fat content; 25 ml of Wijs solution was then added. The flask was sealed and the solution was continuously shaken for 30 min. Also, 20 ml aqueous KI solution (15% v/v) and 100 ml of water were then added to the mixture. The mixture was titrated with 0.1 N Na_2_S_2_O_3_ until the yellow color disappeared. A few drops of starch solution was then added, which changes the solution to blue, and the titration was continued until the blue color disappeared. The volume of spent Na_2_S_2_O_3_ was recorded and represented as *S*. For the control experiment, the titration step was repeated with blank sample and the volume of spent Na_2_S_2_O_3_ is represented as *B*. The IV was calculated using

(3) $$IV=\frac{\left(B-S\right)\times N\;\text{of}\;Na_{2}S_{2}O_{3}\times 12.69}{\text{weight}\;\text{of}\;\text{sample}}$$

#### 2.4.6 Saponification value

The saponification value (SV) of the VCO was determined using the International Union of Pure and Applied Chemistry (IUPAC) method (Rigaudy & Klesney, 1992). About 2.0 g of VCO sample was mixed with 25 ml of 0.5 N ethanolic KOH, and the mixture was boiled for 60 min in a reflux condenser. The mixture was then cooled down to room temperature and subsequently titrated with 0.5 N HCl using 1% phenolphthalein solution as an indicator until the color of the mixture changed from pink to colorless. The volume of spent HCl was recorded and represented as *S*. A similar experiment was repeated with a blank, and the volume of spent HCl was noted as *B*. The SV was calculated using

(4) $$SV=\frac{(B-S)\text{ml}\;\text{of}\;HCl\times 28.05}{\text{weight}\;\text{of}\;\text{sample}(g)}$$

#### 2.4.7 Viscosity

The kinematic viscosity of the VCO samples were measured using Ostwald U‐tube viscometer (Cannon Instruments, USA). The measurements were held in a controlled temperature bath at 25.0°C. The reference liquid was water, where its viscosity (η_*r*_) at 25.0°C is 1.002 cP. The viscosity (η) of the VCO was calculated using

(5) $$\eta =\frac{m\times t\times \eta_{r}}{m_{r}\times t_{r}}$$

where *m* and *t* is the mass and time flow of the VCO and *m*_*r*_ and *t*_*r*_ is the mass and time flow of the water respectively.

#### 2.4.8 Peroxide value (PV)

The PV of the VCO was determined according to the standard IUPAC method (Rigaudy & Klesney, 1992). 5.0 g of VCO was added into a 25 ml acetic acid‐chloroform (3:2) solution, followed by adding 1 ml of saturated KI solution, and the solution was stirred until the oil has been completely dissolved. The solution was then incubated in the dark for 1 hr at room temperature, followed by addition of 75 ml of distilled water. Finally, the solution was titrated with 0.01 N Na_2_SO_3_ with a starch solution as an indicator until the color changes to colorless. The volume of titration was recorded and the PV was calculated using

(6) $$PV=\frac{0.01\times V}{W}$$

where *PV* unit is in milli‐equivalents (m‐eq) of peroxide O_2_ per kg of VCO, *V* is the volume of Na_2_SO_3_ solution (0.01 N), and *W* is the weight of VCO (kg).

#### 2.4.9 Total phenolic content

The total phenolic content (TPC) of the VCO was estimated using Folin–Ciocalteau reagent (Gutfinger, 1981). Polyphenol compounds were extracted from the VCO by dissolving 10.0 g VCO in 50 ml hexane and three successive extractions with 20 ml of 80% methanol. The extract was dried using a rotary evaporator, and the final residue was mixed with 5 ml of 80% methanol. An aliquot (0.3 ml) of the mixture was treated with 1.5 ml of 10‐fold diluted Folin‐Ciocalteau reagent. A volume of 1.3 ml of 7.5% Na_2_CO_3_ was then added into the solution, and it was allowed to stand in the dark at room temperature for 30 min. The absorbance of phenolic content in the solution was recorded using spectrophotometer (Shimadzu, Japan) at 760 nm. The total phenolic content was expressed as GAE per gram of VCO (Nevin & Rajamohan, 2010).

#### 2.4.10 Antioxidant capacity

Antioxidant capacity of the phenolic compounds extracted from the VCO (described in Section 2.4.9) was measured based on its DPPH radical‐scavenging activity according to the reported method (Hatano, Kagawa, Yasuhara, Tasuhara, & Okuda, 1988). In this study, the extracted phenolic compounds (1,000 μl) with concentrations ranging from 0 to 5,000 ppm were added into 1,000 μl methanolic solution of DPPH (50 ppm). The reaction mixture was then vortexed at 40 Hz for 5 min and kept in the dark at room temperature for 30 min. The absorbance of the mixture was measured at 520 nm using single‐beam spectrophotometer (Shimadzu, UV–1800, Japan). Radical scavenging activity (RSA) related to the inhibitory effect of DPPH radical was calculated according to

(7) $$\text{RSA}\left(\%\right)=\left[\frac{A_{\mathrm{control}}-A_{\mathrm{VCO}}}{A_{\mathrm{control}}}\right]\times 100\%$$

where *A*_*control*_ is the absorbance of the control solution and *A*_*VCO*_ is the absorbance of the reaction mixture. We have plotted a curve of *RSA* of DPPH activity against concentration of the VCO, and from the plot we deduced IC_50_ value which was attributed to the concentration of the phenolic compounds extracted of VCO required for 50% RSA.

#### 2.4.11 Metal contents

Acid digestion was used to determine the amount of metals, including lead, copper and iron (Pb, Cu, and Fe), contained in the VCO according the method reported by Ang and Lee (2005). About 0.5 g of VCO was added into 9 ml of freshly‐prepared mixture of HNO_3_ (63%) and HCl (37%) at 1:3 ratio in a digestion flask. The mixture was boiled gently over a water bath at a temperature of 80–90°C for 4–5 hr until the sample had completely dissolved. Once digestion has completed, the mixture was then cooled down to room temperature and filtered through filter paper (Whatman No. 42; 2.5‐μm particle retention). The extract was then evaporated to remove excessive acid, and the volume was topped up to 50 ml with distilled water. The metals in the VCO were quantitatively measured using Nov AA 300 (Analytik Jena, Germany) atomic absorption spectrometer (AAS). The measurement condition was optimized for the determination of the metals with the limit of detection of 1 μg/l. The standard calibration of the metals was measured from 0 to 10 ppm.

#### 2.4.12 Data analysis

In this work, all physicochemical measurements of the VCO and control solutions have been performed at least in triplicate and all data have been analyzed. All results are presented as the average value of the measurements.

## 3 RESULTS AND DISCUSSION

### 3.1 Physicochemical properties

The different extraction methods of VCO, including C&C, FER, DME‐OD, and DME‐SD, resulted in large differences in the yield. The highest yield was given by DME‐OD (47.92%), followed by DME‐SD (40.60%), C&C (20.44%), and FER (9.43%). In Table 1, we show the physicochemical properties, namely the MC, RI, IV, SV, PV, viscosity, and FFA of the VCO extracted by the four different methods. The standard values related to the physicochemical properties and antioxidant capacities of VCO provided by APCC (APCC (Asian Pacific Coconut Community), 2009) are listed for comparison.

**Table 1:** Physicochemical properties of VCO extracted from different methods

Parameters	Extraction method				APCC standard
	C & C	FER	DME‐OD	DME‐SD	
MC (%)	0.15 ± 0.09	0.13 ± 0.06	0.10 ± 0.05	0.17 ± 0.12	≤0.3
RI	1.4543	1.4544	1.4543	1.4544	1.4480–1.4492
IV^a^	0.97 ± 0.08	0.91 ± 0.03	0.61 ± 0.15	0.91 ± 0.03	4.1–11
SV^b^	259 ± 0.74	263 ± 0.30	271 ± 2.79	270 ± 3.63	248–265
PV^c^	2.2 ± 0.3	2.8 ± 0.3	3.4 ± 0.3	4.2 ± 0.3	≤3
η (cP)	50.7 ± 1.0	52.5 ± 1.1	52.4 ± 1.1	48.4 ± 0.9	NA^d^
FFA (%)	0.17 ± 0.06	0.53 ± 0.15	0.30 ± 0.10	0.40 ± 0.10	≤0.2

FER, fermentation; VCO, virgin coconut oil.

^a^ in g I_2_/100 g fats; ^b^ in mg KOH/1 g; ^c^ in m‐eq O_2_/kg; ^d^ NA, not available.

The MC of the VCO in this study was found to be in the range of 0.10%–0.17% (*w/w*), which is within the value recommended by APCC (≤0.3%w/w). This indicates that all of the VCO extracted using C&C, FER, DME‐OD, and DME‐SD methods have low MC, fulfilling the APCC limit. It is noted that the values are slightly higher than that of VCO extracted using fresh‐dry method (0.04%–0.11%) (Mansor, Che Man, Suhaimi, Abdul Afiq, & Ku Nurul, 2012). With their low moisture contents, all of the VCO in this current study is expected to have a long storage life. Notably, the lowest and highest MC is found in VCO extracted by DME‐OD and DME‐SD, respectively. The high MC shown in DME‐SD may be due to the limitation of using sunshine to dry the VCO for a short time (3–4 hr). Drying under sunshine shorter than this period would leave some more water component in the VCO. We expect that the higher moisture content in the oil may give higher percentage of FFA (Che Man, Abdul Karim, & Teng, 1997; Che Man, Suhardiyono Asbi, Azudin, & Wei, 1996).

The RI values of the VCO studied were found to be very narrow from minimum 1.4543 to maximum 1.4544 with a standard deviation of 0.0002. These values were slightly higher (by about 0.006) than the APCC standard range. This deviation is most probably due to the high FFA and FAME contents rather than the purity of the VCO samples. Therefore, we could consider that the certain compositions of FFA and FAME could result in the slightly higher RI of VCO compared with the APCC standard.

The IV of the VCO was found to be between 0.61 and 0.91, much lower than the recommended value by APCC (4.1–11). The lowest IV was measured in VCO extracted using DME‐OD method and the highest IV was detected in VCO produced using C&C method. Since IV indicates the weight percentage of VCO related to unsaturated fatty acids that can absorb halogens such as iodine (Fakhri & Qadir, 2011; Nandi, Gangopadhyay, & Ghosh, 2005), the VCO in this study has a low content of unsaturated fatty acids to bind halogens.

The SV of all the VCO showed high values ranging from 259 to 271 mg KOH/g of fats, while the standard value for SV is 248–265 mg KOH/g fats (Codex, 2001). The highest SV was found in VCO through the dry process, that is, both DME methods, followed by FER and the lowest was found by C&C method. The SV is related to the mean molecular mass of the fats and oils, and it is inversely related to the chain length of the fatty acids fats and oils. This means that the higher the SV, the shorter average chain length of fatty acids. Therefore, VCO extracted by both DME methods tends to possess higher content of the shorter average chain length of fatty acids, in contrast to the VCO from C&C and FER methods. Nevertheless, in general, all the VCO in this study is highly acceptable according to the high MCFA contents.

The PV of the VCO is within 2.2–4.2 m‐eq O_2_/kg, as displayed in Table 1. The PV of VCO extracted using C&C and FER (the wet process) methods is within the APCC recommendation (≤3.0 m‐eq O_2_/kg), whereas that extracted using both DME (the dry process) is slightly higher than the recommended value. Considering that the PV can be used as an indicator of the oxidation or rancidity level of VCO, the low PV of the VCO obtained by the wet process in this study indicates that they are fresh or at early state of oxidation. On the other hand, the high PV of VCO extracted by the dry process is due to oxidation of the substances in the grated coconut meat before the oil is being extracted.

The values for viscosity of the VCO were found to be in the range of 48.4–52.5 cP. FER method gave the highest viscosity (52.5 ± 1.1 cP) followed closely by DME‐OD, C&C, and DME‐SD methods. It is indicated that the viscosity of the VCO is governed by the FFA and FAME composition. It is due to the fact that the viscosity and the laminar flow of VCO vary with the changes in its FFA and FAME composition.

The FFA obtained from VCO this study is in the range of 0.17%–0.53%. The lowest FFA of VCO extracted by C&C, followed by that from DME‐OD and DME‐SD methods. The FFA of these methods are still within the range of the APCC standard (≤0.5%), but VCO extracted by FER has a slightly higher FFA than that of the standard value. The difference in FFA content among the VCO is due to the variation in their processing conditions. We may note the FFA can be used as an indicator of taste and aroma of VCO; thus, the low FFA of VCO in this study suggests that all of their quality is acceptable by the APCC standard. For comparison, VCO produced by integrated wet process has an FFA of 0.13% (Ahmad, Hasham, Aman Nor, & Sarmidi, 2015).

### 3.2 The antioxidant capacity

We summarize polyphenol content and the antioxidant capacity of the VCO as given by TPC and IC_50_ in Table 2. The TPC in the VCO was found to be in the range of 1.16 to 12.54 mg GAE/g oil. The highest TPC (12.54 ± 0.96 mg GAE/g oil) was found in the VCO extracted using FER, followed by DME‐SD, DME‐OD, and C&C methods. It is well known that TPC in oil was strongly affected by the processing methods (Marina, Che Man, & Amin, 2009). Notably, the TPC in the VCO extracted using FER and DME‐SD is much higher compared with that obtained by DME‐OD and C&C methods. This finding may not be so surprising, as it has been suggested that the dry process may destroy some of the phenolic compound in the VCO (Seneviratne & Dissanayake, 2008). In other words, the TPC in VCO by wet process tends to be higher than that of the dry process. Interestingly, the VCO extracted using FER method shows a very high TPC, four times higher than that of the values obtained in the studies reported by Ahmad et al. (2015) or Nur Arbainah (2012), which is about 16.02 and 4.34 mg GAE/g oil, respectively, using the same method of integrated wet process. Our findings revealed that the FER method, a wet process, gave the highest TPC in the VCO.

**Table 2:** Total phenolic content (TPC) and free radical scavenging activity (IC_50_) of VCO produced from different methods

Parameters	Extraction method			
	C & C	FER	DME‐OD	DME‐SD
TPC (mg GAE/g)	1.16 ± 0.05	12.54 ± 0.96	1.56 ± 0.24	8.57 ± 0.36
IC_50_ (mg/ml)	58.71	7.49	104.52	82.52

FER, fermentation; VCO, virgin coconut oil.

We recall that the phenolic compounds in VCO have been determined by Marina, Che Man, Nazimah, and Amin (2008). They are mainly protocatechuic, vanillic, caffeic, syringic, ferulic, and p‐coumaric derivatives, which strongly contribute to the antioxidant capacity of the VCO. The TPC in VCO has been demonstrated to be higher compared with that in the refined coconut oil (Dia, Garcia, Mabesa, & Tecson‐Mendoza, 2005) or the refined, bleached, and deodorized coconut oil (Marina, Che Man, Nazimah, & Amin, 2009). The beneficial effects of the phenolic antioxidants and their high content in VCO make it to be one of the edible oils rich in phenolic compounds. Moreover, VCO with higher TPC is expected to have a higher antioxidant capacity.

As shown in Table 2, the IC_50_ values, which represent the concentration of the VCO needed to reduce 50% of the initial DPPH radicals, is in the range of 7.49 to 104.52 mg/ml. This so‐called radical scavenging activity (RSA) of the VCO is much higher (indicating their lower antioxidant capacity) than other reported VCO of Malayan tall dwarf variety extracted using wet processes (1.24 mg/ml in VCO extracted using FER and 1.66 mg/ml in VCO extracted using chilling and thawing technique, Marina, Che Man, & Amin, 2009). Although all of the VCO should contain hydrogen‐donating capability, this result implies that a significant difference in the antioxidant activity of the VCO depends on the processing condition. In this regard, the VCO obtained using FER shows the strongest antioxidant capacity, followed by C&C and DME methods. Notably, the highest TPC was found to be the VCO extracted using FER method. Therefore, this finding highlights the correlation between TPC and the scavenging activity, and hence the antioxidant capacity. This is in accordance with that reported by Marina, Che Man, and Amin (2009) where the VCO with FER method has the strongest scavenging effect on DPPH and the highest antioxidant activity. Interestingly, VCO obtained through C&C method shows higher antioxidant capacity although its TPC is the lowest. It can be rationalized that cold extraction condition employed in the C&C method may preserve the thermally unstable antioxidant compounds in the VCO.

### 3.3 FAME composition

The FAME compositions of the VCO extracted by the four different methods are shown in Table 3. This fatty acid analysis is essential to provide the information regarding fatty acid distribution in the VCO (Kamariah et al., 2008). In this study, it was found that the fatty acid predominantly contains lauric acid (C12) ranging from 48.40% to 52.84%, which is in agreement with the APCC standard for VCO (45.10%–53.20%) (APCC (Asian Pacific Coconut Community), 2009). The highest lauric acid composition was found in VCO extracted using DME‐OD. This VCO has higher lauric acid composition compared with that of C&C, FER, or even with the integrated wet process (Ahmad et al., 2015; Hamid, Sarmidi, Mokhtar, Sulaiman, & Azila, 2011). Though the variation of the fatty acid compositions can occur during extraction, our findings demonstrate that DME‐OD is an efficient method to produce high lauric acid composition. It is noteworthy that the total MCFA (C6–C12) is 70.1%, 69.6%, 71.3%, and 65.7% for VCO extracted using C&C, FER, DME‐OD, and DME‐SD, respectively. These values are much higher than that found in VCO produced by the integrated wet process 62.6%–63.7%) (Hamid et al., 2011). Overall, the fatty acid analysis suggests that the VCO in this study has a high total MCFA. Consequently, their long‐chain fatty acid (C14–C18) content which is 24.7%, 24.0%, 22.3%, and 19.8%, respectively, is much lower than those in VCO produced by the integrated wet process (29.05%). Another important fatty acid composition is the unsaturated fatty acid in the VCO. As shown in Table 3, the unsaturated linoleic acid C18:1 is in the range of 2.1% to 2.9%, whereas the amount of linoleic acid C18:2 was undetected in the VCO samples. However, the undefined fatty acid which is roughly more than 5.3% may contain some longer chains of unsaturated fatty acid.

**Table 3:** Fatty acid composition of VCO produced from different methods and APCC standard FA for VCO (% area)

Fatty acid	Extraction method				APCC standard
	C & C	FER	DME‐OD	DME‐SD	
C6:0	0.69	0.69	0.78	0.76	0.10–0.95
C8:0	9.67	10.11	10.3	9.77	4–10
C10:0	7.14	7.50	7.37	6.86	4–8
C12:0	52.55	51.30	52.84	48.40	45–56
C14:0	15.37	14.75	14.57	12.93	16–21
C16:0	5.24	5.30	4.62	3.95	7.5–10.2
C18:0	1.16	1.17	0.86	0.80	2–4
C18:1	2.90	2.74	2.21	2.11	4.5–10
C18:2	ND	ND	ND	ND	0.7–2.5
Others	5.28	6.44	6.39	14.42	NA

FER, fermentation; VCO, virgin coconut oil.

### 3.4 Metal contents

The metals contents in the VCO in this study were analyzed using AAS with acid digestion method as described in section 2.4.11. As listed in Table 4, it is found that Cu is in the range of 0.01 to 0.07 ppm, Fe 0.13 to 0.44 ppm, and Pb in the range of 0.03 to 0.07 ppm, respectively. It is interesting to note that Cu and Fe are well known as prooxidants due to their ability to catalyze the disintegration (decomposition) of hydroperoxides in oil into free radicals, whereas Pb, the heavy metal, is known for its toxicity and carcinogenicity (Ali, 2017; Chi, Zuo, & Liu, 2017). Thus, for healthy and safety assurance, the presence of Cu, Fe, and Pb in the VCO should not exceed a certain value. According to APCC standard, the maximum permitted concentration for Cu, Fe, and Pb in VCO should be less than 0.4, 5, and 0.1 ppm, respectively. Based on the data presented in Table 4, those metal contents in VCO of this study do not exceed the recommended APCC limit, ensuring the quality and safety assurance of the VCO for consumption as well as for utilization externally. However, in comparison, Pb level in the VCO is a few times higher than that in vegetable oils (in the range of 0.0060.018 μg/g) (Zhu, Fan, Wang, Qu, & Yao, 2011), but it is much lower than that in sesame oils (0.1250.200 μg/g) (Park et al., 2013).

**Table 4:** The contents of metals (μg/g) in VCO produced from different methods

Metals	Extraction method				APCC standard
	C & C	FER	DME‐OD	DME‐SD	
Pb	0.03 ± 0.01	0.03 ± 0.01	0.06 ± 0.02	0.07 ± 0.05	≤0.1
Cu	0.05 ± 0.01	0.07 ± 0.03	0.01 ± 0.00	0.02 ± 0.01	≤0.4
Fe	0.24 ± 0.06	0.44 ± 0.32	0.14 ± 0.09	0.13 ± 0.01	≤5

FER, fermentation; VCO, virgin coconut oil.

## 4 CONCLUSIONS

In this study, we have investigated the physicochemical properties (moisture content, refractive index, viscosity, iodine value, saponification value, peroxide value, and free fatty acid) of VCO produced in Brunei Darussalam, which were obtained through four different methods, including the wet and dry processes. With the different processing methods, the extraction yield is in the range of 9.4%–47.9%, with the highest oil recovery being obtained from the dry processes (DME methods). We found that most of their physicochemical properties are within the acceptable range or comparable with the recommended values given by APCC (Asian Pacific Coconut Community), 2009. All of the VCO predominantly contains lauric acid as high as 48.40%–52.84% of the fatty acid content with the total MCFA being in the range of 65.7%–71.3%. The phenolic compounds in the VCO were found in a certain amount depending on the processing method, and their DPPH radical‐scavenging activity was obtained to be 7.49–104.52 mg/ml. The metal contents in the VCO are also within the acceptable range of the recommended APCC limit, ensuring the quality and safety assurance of the VCO for consumption as well as for utilization externally. These findings ensure good quality and safety assurance of the VCO produced from the coconut grown in Brunei Darussalam through the different processing methods. Overall, in terms of cost to extract, yield, and quality of VCO, we conclude that the DME‐OD is the most suitable method for mass production of VCO.

## CONFLICT OF INTEREST

None declared.

## ETHICAL STATEMENT

Ethical approval not required because this study did not involve human and animal, but it only involved coconut and coconut milk.
